# Bioelectrical phase angle values in a clinical sample of ambulatory rehabilitation patients

**DOI:** 10.1186/1476-5918-7-14

**Published:** 2008-09-10

**Authors:** Simon M Gunn, Julie A Halbert, Lynne C Giles, Jacqueline M Stepien, Michelle D Miller, Maria Crotty

**Affiliations:** 1 Flinders University Department of Rehabilitation and Aged Care, Repatriation General Hospital, Adelaide, Australia; 2 Flinders Centre for Clinical Change and Health Care Research, Flinders University, Adelaide, Australia; 3 Flinders University Department of Nutrition and Dietetics, Adelaide, Australia

## Abstract

**Background:**

Phase angle (PhA) is derived from the resistance and reactance measurements obtained from bioelectric impedance analysis (BIA) and is considered indicative of cellular health and membrane integrity. This study measured PhA values of rehabilitation patients and compared them to reference values, measures of functional ability and serum C-reactive protein (CRP) levels to explore their utility as a clinical tool to monitor disease progression and treatment efficacy.

**Methods:**

This cross-sectional observational study was conducted on 215 ambulatory rehabilitation patients aged 20 – 94 years. All participants had been hospitalised for a stroke, orthopaedic or other condition resulting in a functional limitation. PhA was derived from BIA analysis and functional ability characterised using the Functional Independence Measure (FIM), timed up and go (TUG) and maximal quadriceps strength (MQS). Serum levels of CRP were also collected.

**Results:**

Stroke patients had the highest PhA (5.3°) followed by elective orthopaedic surgery (5.0°) with the other group (4.3°) significantly lower than both previous categories (p < 0.001). Ambulatory rehabilitation patients' PhA values were dependent on age and sex (p < 0.001), lower than published age matched healthy reference values (p ≤ 0.05) and similar to other hospitalised or sick groups, but also higher than values reported in critically ill patients. Patients with CRP values less than 10 mg.L^-1 ^had significantly (p = 0.005) higher mean PhA values. Furthermore, the highest functional status quartiles had significantly higher PhAs (p ≤ 0.04) for the FIM, MQS and TUG measures.

**Conclusion:**

The results suggest that the phase angles of rehabilitation patients are between those of healthy individuals and seriously ill patients, thereby supporting claims that PhA is indicative of general health status. Phase angles are a potentially useful indicator of functional status in patients commencing an ambulatory rehabilitation program with a normal hydration status.

## Background

Bioelectrical impedance analysis (BIA) is a simple, non-invasive technique primarily used for body composition analysis. Its ease of use and portability has made it a useful tool for evaluating the nutritional state of the elderly in both clinical and research environments. BIA uses body resistance (R) and reactance (Xc) to a flow of alternating electrical current to determine impedance and estimates body composition parameters from regression equations derived against a reference method. Phase angle (PhA) is calculated as the arctangent of the ratio of Xc to R (converted to degrees) with values for the majority of people lying between 3° to 15°[1].

The physiological role of PhA has not yet been well defined, but it is considered a gauge of membrane integrity and intra- and extracellular water distribution. Originally correlated with various physiological variables and used to aid the diagnosis of some metabolic disorders, PhA has more recently shown promise as a prognostic tool and general indicator of health status. Previous investigations have reported a positive relationship between PhA and recovery in critically ill patients [2] as well as survival times in those undergoing hemodialysis [3], or with HIV [4], lung cancer [5] and cirrhosis [6]. Recent studies have also demonstrated that PhA is a strong prognostic indicator in advanced pancreatic [7] and colorectal [8] cancers. This work suggests that PhA may be more sensitive than biochemical markers as a technique to monitor disease progression and treatment efficacy.

This study investigated whether PhA could prove to be an easily measured, non-invasive objective measure of nutritional or general health to be used in conjunction with regular functional performance outcome measures. PhA values of ambulatory rehabilitation patients were compared with healthy reference values, a sensitive serum indicator of low-level inflammation and some measures of functional status commonly used in this population.

## Methods

### Subjects

Two hundred and fifteen participants aged 20 to 94 were recruited from a total of 306 patients referred to a regional rehabilitation service between June 2005 and June 2006. Patients were eligible if they were medically stable, ready for hospital discharge and had rehabilitation goals that required at least 12 sessions. They were ineligible if they lived outside of the health region or were unable to undertake the BIA measurements. All participants had been hospitalised for a stroke, orthopaedic or other condition resulting in a functional limitation and had been referred to the Department of Rehabilitation and Aged Care, Repatriation General Hospital for ambulatory rehabilitation. This project was approved by the Repatriation General Hospital Research Ethics Committee. All experimental procedures, possible risks and benefits were explained to the subjects before their written informed consent was obtained.

### BIA measurements

All anthropometric measurements were taken at ambient temperature with subjects fasted for a minimum of 4 h while wearing minimal, light clothing and with their shoes removed. Body mass was measured to the nearest 0.1 kg with a portable platform scale (Vogel & Halke GmbH & Co.). Knee height was determined to the nearest 0.1 cm using a Ross knee height caliper (Ross Laboratories) and this was used to estimate stature using age, gender and race specific equations [9]. BIA measurements were performed with a QuadScan 4000 instrument (Bodystat Ltd, Isle of Man) which applies a 200 μA current at frequencies of 5, 50, 100 and 200 kHz. Subjects were in a supine position with their arms and legs abducted to ensure that no parts of the body were touching. The first set of electrodes was attached to the dorsal surface of the right wrist between the radial and ulnar processes and directly behind the knuckles on the back of the hand. The second set of electrodes was positioned on the anterior surface of the ankle midway between the medial and lateral malleoli and behind the toes on the top of the foot. The results displayed on the analyser were recorded and then stored for later analysis. The phase angle for the whole body at 50 kHz were calculated from the impedance values using software supplied by Bodystat Ltd.

### Functional measurements

Three assessments of functional status were performed by all participants. The modified Barthel Index (MBI) which is a 10-item index of functional independence, particularly focusing on personal and domestic activities of daily living [10]. The Timed Up and Go (TUG) was used as a fast reliable and valid test for functional mobility and involves the timing of the participant rising from a chair, walking 3 m and returning to a sitting position [11]. The Functional Independence Measure (FIM), a multidimensional functional status tool pertaining to basic life activities, was also measured [12].

Quadriceps strength was independently measured on both legs using a manual muscle tester (model 01160, Lafayette Instrument Co.). Three trials were conducted on each leg and the mean of the two final efforts used to calculate the strength value for that leg. In order to eliminate any bias resulting from a subject's orthopaedic surgery or hemiparesis only the highest mean quadriceps strength value of either the left or right leg was used in further analyses.

### Blood measurements

Serum C-reactive protein (CRP) was assessed from blood samples taken using standard venepuncture techniques. Analyses were performed using a particle-enhanced immunoturbidimetric assay with a functional sensitivity of 1.0 mg.L^-1 ^(coefficient of variation = 1.8%) on a Hitachi 917 Modular P automatic analyser (Hitachi Co., Japan).

### Statistics

The statistical analyses were performed using Statistical Package for the Social Sciences version 12.0.1 (SPSS Inc.). Independent *t*-tests were used to detect possible differences between men and women, while single sample *t*-tests were employed to determine if significant differences existed between measured PhAs and published reference values. ANOVA was used to examine differences between the quartile groups and age categories. Tamhane's T^2 ^post-hoc comparisons were conducted in the event of a significant F-ratio. Pearson correlation coefficients were also calculated between PhA and functional and biochemical variables. The 0.05 level was used for all tests of statistical significance.

## Results

### Descriptive characteristics

The descriptive and functional characteristics of the study participants are contained in Table 1. Fifty three percent of the participants were women and 95% were aged 45 years or more. The men were significantly taller (p < 0.001) and heavier (p < 0.05) than the women, however, there were no significant age or body mass index (BMI) differences between sexes. There were also no significant sex differences in functional ability, with the exception of maximal quadriceps strength where the men were significantly stronger than the women (7.5 kg vs 5.4 kg, respectively; p < 0.001). Thirty one percent (n = 66) of the sample had no more than some mild oedema of the lower limb or fingers.

**Table 1:** Descriptive statistics of the 20 to 94 year old ambulatory rehabilitation patients (n = 215)

	**Men (n = 102)**	**Women (n = 113)**	**Combined (n = 215)**
**Age (yr)**	71.7 ± 12.1 (24 – 94)	71.4 ± 15.5* (20 – 90)	71.5 ± 14.0 (20 – 94)
**Height (cm)**	171.3 ± 6.1 (154 – 186)	159.0 ± 6.2^† ^(146 – 180)	164.9 ± 8.7 (146 – 186)
**Mass (kg)**	77.0 ± 14.3 (40.3 – 132.5)	70.3 ± 21.0^‡ ^(41.1 – 156.0)	73.5 ± 18.4 (40.3 – 156.0)
**BMI (kg/m**^2^**)**	26.2 ± 4.2 (15.2 – 39.1)	27.6 ± 7.0* (13.6 – 53.3)	26.9 ± 5.9 (13.6 – 53.3)
**% BF**	28.9 ± 5.4 (15.1 – 44.1)	42.7 ± 6.8^‡ ^(22.6 – 57.6)	36.2 ± 9.3 (15.1 – 57.6)
**Length of stay (days)**	14.8 ± 17.1 (3 – 120)	13.9 ± 10.5* (3 – 65)	14.4 ± 14.0 (3 – 120)
**TUG (sec; n = 213)**^§^	31.7 ± 29.2 (7.7 – 207.6)	33.7 ± 21.8* (8.2 – 131.6)	32.7 ± 25.5 (7.7 – 207.6)
**MBI (n = 214)**^§^	91.9 ± 6.4 (71 – 100)	92.7 ± 6.7 (56 – 100)	92.3 ± 6.6 (56 – 100)
**FIM (n = 210)**^§^	109.0 ± 10.4 (68 – 124)	107.8 ± 10.5 (75 – 124)	108.4 ± 10.4 (68 – 124)
**Max Quad strength (kg)**	7.5 ± 3.5 (1.5 – 16.8)	5.4 ± 2.8^‡ ^(0.4 – 13.1)	6.4 ± 3.3 (0.4 – 16.8)

All values are $\bar{\text{X}}$ ± SD; range in parentheses; %BF = percent body fat; TUG = Timed Up-and-Go; MBI = modified Barthel Index; FIM = Functional Independence Measure

* Non-significant; ^† ^p < 0.001; ^‡ ^p < 0.05

^§ ^Subjects with missing data were excluded from the corresponding analyses.

### Clinical characteristics

The 215 patients had a median hospital stay of 9 days prior to beginning their ambulatory rehabilitation program and entered the study on discharge from hospital. Stroke accounted for 37% of the participants' diagnosis on admission while elective orthopaedic surgery (total knee replacement) and a variety of other conditions associated with an emergency admission and deconditioning (eg. intensive care stay, fractured neck of femur and other orthopaedic or neurological conditions) were experienced by 20 and 44% of the sample, respectively. Stroke patients had the highest PhA (5.3°) followed by those admitted for elective orthopaedic surgery (5.0°) with the other group (4.3°) significantly lower than both of the previous two categories (p < 0.001). There were no age or sex differences in the composition of these diagnostic groups.

### Phase angle

The PhA data are displayed by age and sex in Table 2 and show that for all age groups the men had significantly higher values than the women (5.1 vs 4.5, respectively; p < 0.001). While the PhA value was also higher for men than women in all age groups containing similar numbers of subjects (i.e. aged 45 yr onwards) this difference was only statistically significant in the 55 – 64 and 65 – 74 yr categories (p < 0.05 and p < 0.001, respectively). The combined data show a predominantly decreasing trend with age (i.e. 6.2° for the youngest age group and 4.4° for the oldest). Similar trends prevailed when the data were divided into groups younger than 80 yrs and 80 yrs and older (Table 2). There was a significant (p < 0.001) age effect for the combined groups, however, although the younger men (i.e. < 80 yr) had significantly higher values than the corresponding women, there was no sex based differences in subjects aged 80 yrs and older.

**Table 2:** Phase angle data by age and sex groups (n = 215)

**Age Group (yr)**	**Men**	**Women**	**Combined**
**Combined**	5.1° ± 1.2 (102) [2.4–8.4]	4.5° ± 0.9^† ^(113) [2.3–6.8]	4.8° ± 1.1 (215) [2.3–8.4]
**20 – 24**	8.4° (1)	5.2° ± 0.1 (2) [5.1–5.2]	6.2° ± 1.9 (3) [5.1–8.4]
**25 – 34**	N/A	5.5° ± 0.7 (3) [4.7–6.0]	5.5° ± 0.7 (3) [4.7–6.0]
**35 – 44**	4.8° (1)	5.4° ± 0.8 (3) [4.6–6.1]	5.3° ± 0.7 (4) [4.6–6.1]
**45 – 54**	6.1° ± 1.3 (8) [3.6–7.8]	5.6° ± 0.9* (9) [4.0–6.8]	5.8° ± 1.1 (17) [3.6–7.8]
**55 – 64**	5.5° ± 1.2 (20) [2.9–7.3]	4.4° ± 0.9^‡ ^(10) [2.3–5.6]	5.1° ± 1.3 (30) [2.3–7.3]
**65 – 74**	5.5° ± 0.9 (18) [3.1–6.8]	4.6° ± 0.8^† ^(25) [2.9–6.4]	5.0° ± 0.9 (43) [2.9–6.8]
**75 +**	4.6° ± 0.9 (54) [2.4–6.2]	4.3° ± 0.8* (61) [2.3–6.0]	4.4° ± 0.9 (115) [2.3–6.2]
**< 80 yrs old**	5.3° ± 1.2 (76) [2.9–8.4]	4.7° ± 0.9^† ^(71) [2.3–6.8]	5.0° ± 1.1^§^(147) [2.3–8.4]
**≥ 80 yrs old**	4.3° ± 1.0 (26) [2.4–6.0]	4.2° ± 0.8* (42) [2.3–5.4]	4.3° ± 0.9 (61) [2.3–6.0]

All values are $\bar{\text{X}}$ ± SD; *n *in parentheses; range in brackets.

* Non-significant; ^‡ ^p < 0.05; ^† ^p < 0.001 for comparisons across sex; §p < 0.001 for the comparison across age

**Figure 1:**
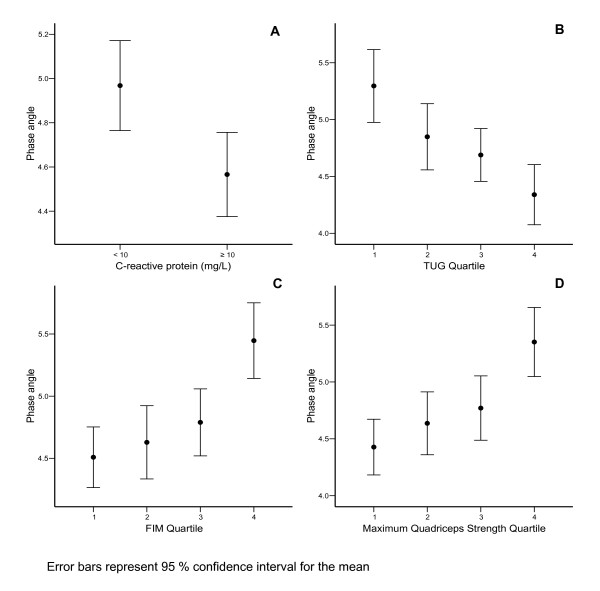
Phase angle by C-reactive protein classification (A), Timed Up-and-Go (B), Functional Independence Measure (C) and maximum quadriceps strength (D) quartiles (error bars represent ± 95% confidence interval of the mean).

Phase angle showed modest but significant (p < 0.01) correlations with maximum quadriceps strength (0.3), FIM (0.3), age (-0.4) and length of acute hospital stay (-0.3). However, there was no significant association with CRP (0.1), BMI (0.1), MBI (0.1) or TUG (0.1). These correlations also tended to be higher in men compared with women (data not shown). Further analyses revealed that patients with CRP values less than 10 mg.L^-1 ^had significantly (p = 0.005) higher mean PhA values (5.0°) than those with CRP greater than 10 mg.L^-1 ^(4.6°; Figure 1). Furthermore, when the sample was divided into quartiles based on the various functional measures, the fourth quartile (i.e. highest values) had significantly higher PhAs than the other three quartiles (p ≤ 0.04) for the FIM and quadriceps strength measures and a significantly lower PhA than the first quartile (p ≤ 0.001) for TUG (Figure 1). These trends remained when the confounding influence of sex was removed. There was no significant difference between any quartile for MBI. PhA also showed a generally decreasing trend with length of acute hospital stay where the fourth quartile (longest stay) had significantly lower values than the first (shortest stay: p = 0.001) and third quartiles (p = 0.03).

**Figure 2:**
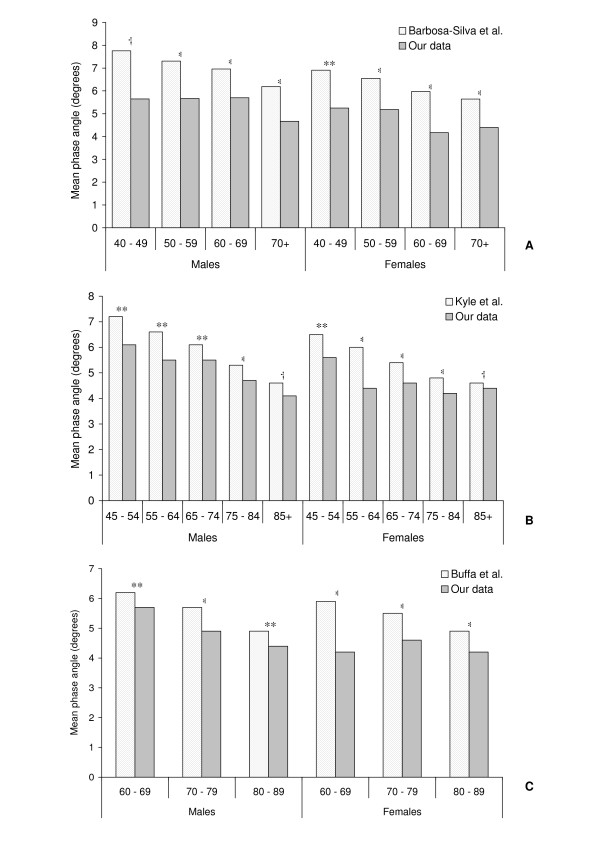
Age-wise comparisons of published phase angle reference values against data obtained from 215 ambulatory rehabilitation patients aged 20 to 94 years.

Our phase angle values were compared to published data in healthy subjects. Due to our small number of subjects in the younger age groups (20 – 24 yr; n = 3, 25 – 34 yr; n = 3 and 35 – 44 yr; n = 4) these comparisons were limited to participants aged 40 yr and over. Compared with various healthy reference ranges, our subjects consistently displayed lower phase angle values (Figure 2). All of these were statistically significant (p < 0.05) except for the 40 – 49 yr males of Barbosa-Silva et al. [13] and the 85+ yr age group of Kyle et al. [14]. Further comparison with the work of Buffa et al. [15] yielded similar results with our phase angles significantly lower for both men and women across all of their reported age groups (Figure 2).

## Discussion

This study clearly shows age and sex related differences exist for PhA values in an ambulatory rehabilitation population. This inverse relationship between age and PhA also supports previous work in healthy subjects [13-17]. The reduced PhA values in the elderly have been suggested to reflect the decrease in general health and physical function associated with ageing [13]. Since PhA is influenced by the ratio of intracellular to extracellular water, the lower values seen in older subjects are thought to reflect a reduction in skeletal muscle, and hence intracellular water, which may also be compounded by increased oedema (extracellular fluid accumulation) with ageing.

The sex-related differences exhibited by our subjects also confirm the findings of a number of previous studies [13,14,16,18]. PhA increases with body cell mass and since males generally have a higher amount of fat free mass (and hence, skeletal muscle) they also have higher PhA values than females.

Phase angle is considered one of the best indicators of cellular health where a higher value reflects stronger cell membrane and better cell function [1,19]. Figure 2 shows that the PhAs reported in studies on healthy subjects are clearly higher than the age matched values observed in this study. Our data are significantly lower than all of the values reported by Buffa et al. [15] for healthy elderly subjects and the majority of those reported by Barbosa-Silva et al. [13] and Kyle et al. [14]. Only three age group comparisons were not significant. Our sample only had two men corresponding to Barbosa-Silva et al. [13] 40 – 49 yr age range and the subsequent lack of statistical power decreased the validity of this comparison. However, both the men and women in the 85+ yr age group of Kyle et al. [14] were not significantly higher than our corresponding subjects. This is possibly a result of the natural agewise decline in PhA bringing these two groups closer together, such that our sample of the rehabilitation population are not significantly different from those people in this age range that are considered "healthy". This trend supports previous comparisons of PhA in HIV patients that showed significant differences between the stable and critically ill [20], and surviving and non-surviving patients [4], suggesting that PhA may be an indicator of disease progression and long term survival. Studies conducted on a small number of critically ill patients by Zarowitz et al. [2] reported a very low mean value of 3.1°. Slightly higher values were found by Mattar et al. in acute respiratory distress syndrome sufferers (3.9°) [21] and critically ill septic patients (4.2°) [1]. Comparisons with values reported in the literature are difficult because between group variation in R values due to hydration or oedema status will affect the PhAs obtained. Nevertheless, these findings indicate that the PhA results for ambulatory rehabilitation patients sit somewhere between the critically ill and healthy individuals, and that the PhA may be used as a marker of general health status and possibly disease progression.

The correlations between PhA, CRP and functional measures were modest. While BIA is considered a valid marker of general health [1,19], the strength of these associations imply that these measures are probably representative of different aspects of health status. Considering that the heterogeneous nature of our sample has also reduced these correlations somewhat, further examination of the group or quartile trends does provide some evidence of the associations between these measures. A closer look at our patients with abnormal values for CRP (> 10 mg/L) showed they had lower PhAs than patients with normal values for this biochemical marker (Figure 1). This study also highlights that a higher quartile ranking on FIM and maximum quadriceps strength were associated with higher PhA values as were lower quartile (faster) TUG results (Figure 1). A similar positive relationship between PhA and muscle strength values (i.e. hip adduction and shoulder abduction) has been reported by Selberg & Selberg [6]. This suggests that lower PhA are associated with poorer functional status and CRP results.

The limitations of this study include the heterogeneous nature of the population, modest sample size and the variability of the BIA technique. Due to the nature of ambulatory rehabilitation our sample contained patients with a wide range of conditions and ages, which may obscure more definitive interpretation of the data. The relatively modest sample size may also have contributed to this. The variability of the BIA technique is related to a number of factors. Our participants were measured using a standardised protocol that controlled for their body position during measurement (reducing any major disturbances of water distribution) as well as food and fluid consumption (~4 hr fasted). The physical activity levels of our patients were inherently quite low and while none exhibited excessive levels of visible oedema there was most likely some variation in hydration status within the sample.

## Conclusion

This study has shown that in a heterogeneous sample of ambulatory rehabilitation patients, PhA values are dependent on age and sex. While these values are lower than published age matched healthy reference values and similar to other hospitalised or sick groups, they are higher than values reported in critically ill patients. Furthermore, we found lower PhAs are associated with poorer functional status and serum inflammatory marker results. Our work supports claims made in healthy populations that PhA is indicative of general health status and shows promise as an indicator of clinical outcome. More work, particularly longitudinal studies and vector analysis to discriminate between possible spurious soft tissue mass differences due to hydration and true soft tissue mass differences is required to explore the utility of phase angle for clinicians, however, it is easily measured and may potentially assist in the identification of patients at risk of poorer outcomes.

## List of abbreviations

BIA: Bioelectrical impedance analysis; BMI: Body mass index; CRP: C-reactive protein; FIM: Functional independence measure; MBI: Modified Barthel index; MQS: Maximal Quadriceps strength; PhA: Phase angle; R: Resistance; TUG: Timed up and go; Xc: Reactance

## Competing interests

The authors declare that they have no competing interests.

## Authors' contributions

MC, MDM, JAH & LCG conceived the study and secured the research funding. JMS helped recruit the subjects and collect the data. SMG & LCG analysed and interpreted the data. SMG wrote the original draft of the manuscript which was then reviewed and edited by all authors.
